# The Impact of Microbial Ecology and Chemical Profile on the Enhanced Biological Phosphorus Removal (EBPR) Process: A Case Study of Northern Wastewater Treatment Works, Johannesburg

**DOI:** 10.3390/ijerph110302876

**Published:** 2014-03-10

**Authors:** Ilunga Kamika, Martie Coetzee, Bhekie Brilliance Mamba, Titus Msagati, Maggy N. B. Momba

**Affiliations:** 1 Department of Environmental, Water and Earth Sciences, Faculty of Science, Tshwane University of Technology, Arcadia Campus, Private Bag X680, Pretoria 0001, South Africa; E-Mails: alainkamika2@yahoo.com (I.K.); CoetzeeMAA@tut.ac.za (M.C.); 2 Department of Applied Chemistry, University of Johannesburg, P.O. Box 17011, Doornfontein 2028, South Africa; E-Mails: bmamba@uj.ac.za (B.B.M.); tmsagati@uj.ac.za (T.M.)

**Keywords:** wastewater, EBPR, polyphosphate-accumulating organisms, glycogen-accumulating organisms, volatile fatty acids

## Abstract

The impact of polyphosphate-accumulating organism (PAO) and glycogen-accumulating organism (GAO) populations as well as of the chemical profile on the performance of Unit-3 (open elutriation tanks) and Unit-5 (covered elutriation tank) of the City of Johannesburg Northern Wastewater Treatment Works was determined. Physicochemical parameters of wastewater samples were measured using standard methods. Bacterial diversity was determined using 16S rRNA gene amplicon pyrosequencing of the variable region V1-3. Results showed soluble COD concentrations from settled sewage for Unit-3 at 192.8 mg COD/L and for Unit-5 at 214.6 mg COD/L, which increased to 301.8 mg COD/L and 411.6 mg COD/L in the overflow from elutriation tanks and decreased to 170.9 mg COD/L and 256.3 mg COD/L at the division boxes, respectively. Both long-chain volatile fatty acids (heptanoic acid, isobutyric acid, 3-methylbutanoic acid, pentanoic acid, 4-methylpentanoic acid, methylheptanoic acid) and short-chain volatile fatty acids (acetic acid, propionic acid, isobutyric acid) were present within concentration ranges of 17.19 mg/L to 54.98 mg/L and 13.64 mg/L to 87.6 mg/L for Unit 3 and 38.61 mg/L to58.85 mg/L and 21.63 mg/L to 92.39 mg/L for Unit 5, respectively. In the secondary settling tanks, the phosphate-removal efficiency in Unit-5 appeared to be slightly higher (0.08 mg P/L) compared to that of Unit-3 (0.11 mg P/L). The average DO concentrations (2.1 mg/L and 2.2 mg/L) as well as the pH values (pH 7 to pH 7.5) were found to be slightly higher in Unit-5 in the aerobic zones. The high presence of PAOs in the bioreactors (Unit-5: *Dechloromonas* (14.96%)*, Acinetobacter* (6.3%)*, Zoogloea* (4.72%) in the anaerobic zone and *Dechloromonas* (22.37 %) in the aerobic zone; Unit-3: *Dechloromonas* (37.25%) in the anaerobic zone and *Dechloromonas* (23.97%) in the aerobic zone) confirmed the phosphate-removal efficiencies of both units. Negligible GAOs were found in the aerobic zones (*Defluviicoccus* spp.: 0.33% for Unit-5 and 0.68% for Unit-3) and in the anaerobic zones (*Defluviicoccus*: 9.8% for Unit-3). The high microbial diversity and a negligible percentage of GAOs in Unit-5 could contribute to its high phosphate-removal efficiency, although results did not indicate statistically significant differences between the unit with a covered elutriation tank (Unit-5) and that with open elutriation tanks (Unit-3).

## 1. Introduction

During the past century, the ever-growing population and industrialisation, as well as rapid urbanisation have resulted in an increase in environmental pollution. This environmental damage has become a global issue due to the public health concerns associated with these pollutants once the polluted wastewater is discharged into receiving water bodies [1]. However, the long-term and wider issues would persist since anthropogenic sources are placing pressure on larger resource bases (water, soil, air), which support our life [2]. 

In South Africa in particular, water pollution is one of the most debated issues due to the rapid industrialisation. South Africa is considered to be a water-stressed country with just over 1,200 m^3^ of fresh water available for each person per year for a population of approximately 49.99 million [3]. However, in terms of water resources, the domestic and industrial effluents generated occupy the second position (with 14% originating from these water sources, 77% from surface water and 9% from groundwater) [4], and currently constitute a major source of chemical and microbial pollution of South Africa’s water sources [5]. Of these chemical pollutants, phosphorus is regarded as a major concern since cyanobacteria, mainly responsible for eutrophication, are capable of fixing molecular nitrogen from the atmosphere, thus eliminating the requirement for ammonia-nitrogen (NH_3_-N) or nitrate-nitrogen (NO_3_-N). Eutrophication, known as excessive microbial growth, results in reduced transparency, reduced photosynthetic activity, depletion of oxygen, production of toxic compounds and loss of plant and animal species [6]. Korstee and co-authors [7] reported that no eutrophication occurs when the phosphorus concentration is reduced to between 8 µg/L and 10 µg/L, even at nitrogen concentrations of as high as 4 mg/L to 5 mg/L. Generally, municipal wastewater systems are the major sources of phosphorus when compared to other sources of water pollution [8]. Considering this increasing and persistent situation, it is imperative to find a way to protect the aquatic environment. 

Activated sludge processes are widely used to reliably and efficiently remove degradable carbon (BOD5) and suspended solids from wastewater [6]. Several modifications of this process have been developed and configured to remove nutrients such as nitrogen (N) and phosphorus (P) in an effort to slow eutrophication in the receiving water bodies [9]. These modifications often exploit the metabolic capabilities of naturally-occurring microorganisms in the sludge. Biological phosphorus removal in wastewater treatment systems using the enhanced biological phosphorus removal (EBPR) process is increasingly being used as an alternative to chemical precipitation processes [8]. 

In view of the above, the advantages of EBPR are the exploitation of the metabolic capacities of naturally-occurring activated sludge microorganisms such as polyphosphate-accumulating organisms (PAOs) which assist in slowing down eutrophication. Another advantage is that the EBPR process can be implemented with relative ease in the activated sludge wastewater treatment systems. This process is achieved by recirculating sludge through anaerobic and anoxic/aerobic conditions, and directing the influent, usually rich in volatile fatty acids (VFA) such as acetate, propionate, to the anaerobic stage. The VFA promotes the enrichment of the system with PAOs which accumulate and store phosphate in excess of their normal metabolic requirements. These organisms also take up and polymerise inorganic phosphate (Pi) to produce polyphosphate (polyP) when they have access to oxygen by providing excess Pi removal in the systems [10]. Carbon transformations involving accumulation and the utilisation of poly-β-hydroxyalkanoates (PHA) and glycogen also have been reported to be central to EBPR [10]. During the subsequent aerobic stage of the EBPR system, the accumulated PHA is utilised for growth and replenishment of the polyP and glycogen pools. 

Although the EBPR process is regarded as an eco-friendly and cost-effective process for the removal of phosphorus, this process is also prone to apparent instability and unreliability [11]. It has been reported that the deterioration and even failure of the EBPR performance can be due to several environmental and operating factors [12]. Excessive aeration may decrease the level of intracellular polymers (PHA and glycogen), thus affecting the phosphorus-removal performance [8]. The intrusion of nitrate or nitrite in the anaerobic zone has also been reported to contribute to the deterioration of the EBPR activity [13].

Besides the above factors, the appearance of GAOs in the EBPR process has been reported to be the main cause of the deterioration in the performance of this process because GAOs compete for substrate VFA with the PAOs, which are responsible for the removal of phosphorus [11]. Similar to the PAOs, the GAOs are also capable of storing VFA as PHA under anaerobic conditions. In contrast, the latter microbial group does not store polyphosphate and uses the intracellular glycogen as both energy and carbon sources for VFA uptake without exhibiting the typical anaerobic P-release and subsequent aerobic P-uptake of the PAOs [10,14]. Owing to the need for better performance of the EBPR process, GAOs are seen as undesirable microorganisms, because they compete with PAO for VFA and do not contribute to the biological removal of phosphorus [15]. 

Despite extensive research spanning more than four decades, the identity of polyP-accumulating organisms is still a matter of debate. This is because to date no organism that has been isolated has been reported to be primarily responsible for carrying out all of the characteristics of carbon and phosphorus transformations associated with EBPR. In the past, *Acinetobacter* spp. were believed to be primarily responsible for polyP accumulation [8,16]. Recent studies have reported that the latter species are not true EBPR organisms and other polyP-accumulating organisms have been identified in full-scale activated sludge systems [15,17]. However, the inability to isolate the responsible microorganisms and to verify the biochemical metabolism appears to be a limitation to gaining a better understanding of the metabolic pathways used by these microorganisms and the characterisation of the entire microbial ecology of the systems [13]. To date, there is no report of any pure culture or fully characterised co-culture capable of performing the anaerobic and aerobic biochemical operations conducive to EBPR; this was believed to be due to their slow growth rates or the inadequacy to mimic their native habitats [18]. While it remains a challenge to identify and isolate the PAOs or GAOs in pure cultures, the disadvantages and inherent biases associated with the microbial community analysis in EBPR using traditional cultivation techniques are well-documented [8]. Compared to traditional cultivation techniques, culture-independent molecular techniques appear to be more successful for the identification of PAOs or GAOs and therefore the characterisation of the microbial ecology of the EBPR systems. 

Since every wastewater treatment system is unique, due to the type of influent, design and other environmental parameters, more research on EBPR is required to fully elucidate the biochemical mechanisms responsible for the cycling of carbon and phosphate by the PAOs. The present study therefore investigated the population dynamics in EBPR activated sludge of Johannesburg Northern Wastewater Treatment Works in order to provide links between the community structure and the performance of the system. Such information will enable a more rational design and operation of EBPR systems, which could lead to increased process stability and efficiency. 

## 2. Materials and Methods

### 2.1. Study Area and Sample Collection

Wastewater samples were collected twice a month from November 2011 to August 2012 from Unit-3 and Unit-5 of Northern Wastewater Treatment Works, Johannesburg. The wastewater samples were collected in 500 mL sterile bottles and immediately placed in a cooler box for transportation to the laboratory for physicochemical analyses and microbial diversity studies. Johannesburg Water’s Northern Wastewater Treatment Works is by far the largest domestic wastewater treatment plant in South Africa. It treats 400 Mega litres *per day* (ML/d) of domestic wastewater from the north of the Hillbrow ridge and includes Alexandra, Randburg, Sandton and some regions in Midrand and Roodeplaat [19]. Northern Wastewater Treatment Works is situated next to the N14, in the Diepsloot area, and has five separate treatment units. This study focused on only two units, namely Unit-3 and Unit-5. 

**Figure 1:**
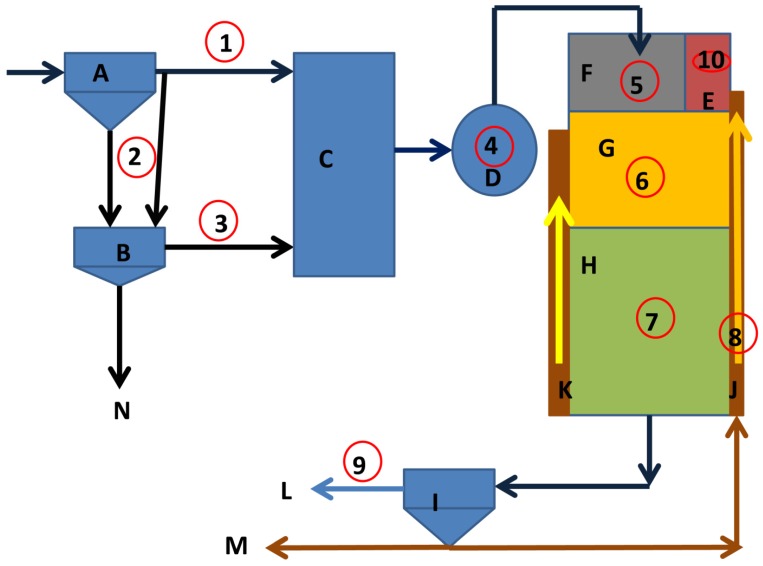
Comparison of the characteristics of Unit-3 and Unit-5 at the Northern Wastewater Treatment Works, Johannesburg.

Notes: **Legend**: **Unit-3**: **A**: Primary settling tanks (2×); **B**: Elutriation tank (2 open tanks). Sludge from PST is pumped into one tank; retention time of 4-6 days. Waste out with settled sewage from PST. Other tank is then filled up; **C**: Balancing tank; **D**: Division box, outflow from balancing tank; **E**: Pre-anoxic tank; receives the RAS; **F**: Anaerobic zone; receives outflow from balancing tank; **G**: Anoxic zone; receives recycle from aerobic zone; **H**: Aerobic zone; **I**: Secondary settling tank; 3 suction lift tanks (difficult to control); **J**: Return activated sludge (RAS) from the bottom of the SSTs; **K**: Recycle stream from aerobic to anoxic zone; **L**: Water effluent from SST goes to disinfection and river; **M**: Waste activated sludge; wasted directly from aeration tank; **N**: Anaerobic digestion; **Unit-5** (design is similar to that of Unit-3 with slight modifications): **B**: Elutriation tank (1×) is covered; receives sludge from PST; retention time of 4 days. The VFA is washed out with settled sewage from the PST; **D**: Division box; outflow from balancing tank. Some turbulence may ingress oxygen; **F**: Anaerobic zone; receives outflow from balancing tank. Excessive “bubbling” where water enters the zone; **I**: Secondary settling tank; mechanically scraped tanks (3×) remove sludge better.

Unit-3 is designed to receive 150 ML/d. The screened and de-gritted wastewaters flow into two PST tanks. The settled sewage flows to a balancing tank. The effluent from the balancing tank flows to a division box, where the flow is divided into three reactors (called Bioreactors 1, 2, and 3). The flow rate into each reactor is between 45 ML/d and 46 ML/d. The inflow is split between the anaerobic zone (90% of the flow) and the pre-anoxic zone (10% of the flow). The biological nutrient removal process consists of the four-stage configuration, internationally recognised as one of the most successful configurations. The unit utilises a side-stream fermentation process to aid in the generation of the VFAs required to achieve biologically enhanced phosphate removal (Figure 1). 

The latest addition to the works is Unit-5, which is designed to receive 50 ML/d. The settled sewage from the two PSTs flows to a balancing tank and subsequently to a division box and ultimately to one biological reactor similar to those used in Unit-3. The main difference between the two units is the fermentation process. Unit-3 is batch-operated and has two mixing tanks and two open elutriation tanks, while Unit-5 is operated on a continuous basis and has one mixing tank and one covered elutriation tank. The data given in Figure 1 provide a brief comparison of the design and operating conditions of the two units. 

### 2.2. Chemical Reagents

Volatile fatty acid standards were purchased from Sigma Aldrich (St. Louis, MO, United States of America), while anhydrous magnesium sulphate (AR grade) was purchased from Merck (Darmstadt, Germany). The solvent methyl tert-butyl ether (MTBE) was obtained from Sigma Aldrich.

### 2.3. Physicochemical Profile of the EBPR

Physicochemical parameters were determined using the standard methods [20]. To remove biomass and other suspended solids, wastewater samples were filtrated using Whatman No. 1 filter papers. The profile of the filtered samples was determined in terms of COD, DO, pH, phosphate, nitrate and VFAs (acetic acid, isovaleric acid, 4-methylvaleric acid, heptanoic acid, isobutyric acid, propionic acid, valeric acid, butyric acid, methyl hexanoate and methyl heptanoate). The COD concentration was measured using closed reflux methods as described in standard methods [20], while the pH and the DO were analysed using a pH probe (Model: PHC101, HACH, Düsseldorf, Germany) and DO probe (Model: LDO, HACH), respectively. To determine the VFAs, samples were acidified to pH 2 using suprapure nitric acid (65% v/v). A 1 mL aliquot was shaken together with 1 mL of MTBE for approximately 10 min, and the ether phases were quantitatively transferred to 4-mL flasks, where a small amount of anhydrous sodium sulphate was added. A series of VFA standards for calibration curves were prepared by taking aliquots from the stock solution and diluting with MTBE in order to make a final concentration ranging from 1 mM to 100 mM.

### 2.4. Microbial Ecology

#### 2.4.1. Isolation of DNA of the Microbial Species

The DNA was isolated from the cells growing in suspension as reported by Ozutsumi *et al.* [21] with slight modifications. Briefly, the cell pellets harvested by centrifugation of 2 mL of the wastewater samples at 10,000 × g for 5 min at 4 °C were re-suspended in 1× TE buffer (pH 8.0). The suspensions were well mixed and bacterial DNA was extracted with the ZR Fungal/Bacterial DNA Kit^TM^ (Zymo Research, Pretoria, South Africa) according to the procedures provided by the manufacturer.

#### 2.4.2. PCR Amplification

The PCR reaction was performed on the extracted DNA samples using universal degenerate primers 27F and 1492R shown in Table 1. Each PCR reaction contained 5 µL of 10× Taq buffer, 2 mM MgCl_2_, 1.5 U Super-Therm Taq DNA polymerase (Fermentas, Vilnius, Lithuania), 0.25 mM dNTPs, 0.1 µM of each primer, 1 µL of extracted DNA and nuclease-free water up to the final reaction volume of 50 µL. The PCR cycle started with an initial denaturation step at 94 °C for 10 min. This was followed by 30 cycles of denaturation at 94 °C for 1 min, annealing at 58 °C for 1 min and extension at 72 °C for 1 min, and a final extension at 72 °C for 5 min, followed by cooling to 4 °C. The PCR product (10 µL) was analysed using 1% (m/v) agarose gel (Merck, SA) stained with 5% of 10 mg/mL ethidium bromide (Merck, SA) and the correct band size (approximately 1,500 bp) was excised. To amplify variable regions (V1-3) of the bacterial 16S rRNA gene, the DNA was recovered from the gel slices by using the GeneJET™ gel extraction kit (Fermentas); thereafter, it was re-amplified with primers A1.4 and B1 [22] as shown in Table 2. These primers contained the appropriate adaptor and barcode sequences that were necessary for running the samples on the GS-FLX-Titanium (Roche). The PCR reaction was analysed as described previously, but with an annealing temperature of 50 °C as reported by Tekere *et al.* [22]. The entire PCR product was loaded onto a 1% agarose gel and the correct band size (500 bp to 600 bp) was excised from the gel and subsequently purified as previously mentioned. 

The DNA concentrations were quantified by using a NanoDrop® spectrophotometer (Nanodrop2000, Thermo Scientific, Japan). The samples were pooled at equal concentrations of the filtration and biofilm samples. The pooled samples were sequenced on the GS-FLX-Titanium series (Roche) at Inqaba Biotechnology Industries, Pretoria, South Africa. 

**Table 1:** Primers used in this study.

Name	Sequence	Reference
27F	5’AGRGTTTGATCMTGGCTCAG3’	Tekere *et al.* [22]
1492R	5’GGTTACCTTGTTACGACTT3’	
A1.4	5’ **CGTATCGCCTCCCTCGCGCCATCA**	
	tctctatgcgAGRGTTTGATCMTGGCTCAG3’	
B1	5’CTATGCGCCTTGCCAGCCCGCTCAG	
	GTATTACCGCGGCTGCTG3’	

**Table 2:** Summary of pyrosequencing data from the wastewater samples.

Sequence	Variable Region (V 1-3) to Be Amplified		
	Elutriation tanks	Anaerobic zone	Aerobic zone
Number of sequences	2,417	2,436	2,098
Total length of sequences (bp)	617,990	671,243	579,638
Average length of sequences (bp)	200	120	180

Sequences of not less than 100 pb were classified (Table 2) using the online Ribosomal Database Project (RDP) naive Bayesian Classifier, Version 2.4 of December 2012, which is assigned to the taxonomical hierarchy: RDP training set 10, based on nomenclatural taxonomy and *Bergey’* *s Manual* [22] with a confidence threshold of 95%.

#### 2.4.3. Statistical Analyses

The data were statistically analysed using the Stata computer software (version: STATA V10, STATA Corp. LP, Texas, TX, USA, 2009). The difference between the physicochemical parameters and also between the microbial abundance during the experimental study was determined by using the one-way analysis of variance (ANOVA). The interpretation was performed at a 95% two-sided confidence interval. 

## 3. Results and Discussion

### 3.1. Physicochemical Parameters

**Figure 2:**
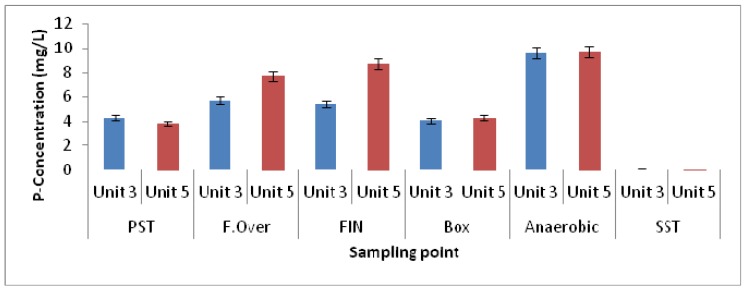
Orthophosphate concentration in wastewater samples collected from different zones of both Unit-3 and Unit-5 of the Northern Wastewater Treatment Works (PST: primary settling tanks; F.Over: overflow elutriation tanks; FIN: inside elutriation tanks; SST: secondary settling tanks).

Both Unit-3 and Unit-5 performed excellently with regard to nutrient removal (phosphate, COD and nitrogen). However, Unit-5 performed slightly better than Unit-3. The average orthophosphate concentrations were 0.11 mg P/L and 0.08 mg P/L, respectively, in wastewater samples collected from Unit-3 and from Unit-5 (Figure 2). Both units have a similar design and construction and currently use the Johannesburg Water process configuration for biological nutrient removal. They make use of a side-stream fermentation process to generate VFA, which improves phosphate release in the anaerobic zone. As stated above, there are, however, two major differences between the fermentation processes in the two units. In Unit-5, the newer plant, the elutriation tank is covered and the sludge is pumped continuously into the tank, whereas the elutriation tanks in Unit-3 are not covered and the sludge from the PSTs is pumped on a batch basis, alternately to two elutriation tanks. For the rest of the process, the operations are fairly similar (see Figure 1).

To evaluate the efficiencies of the fermentation process in the two units, the concentrations of the volatile fatty acids formed in the elutriation tanks of the two units were determined. At the same time, the soluble chemical oxygen demand (SCOD) concentration was also determined, as it gives an indication of the solubilisation process of organic materials in the elutriation tank. The SCOD is composed of the unbiodegradable COD and the readily biodegradable COD. It has been reported that the unbiodegradable soluble COD fraction of settled sewage varies for domestic wastewater in South Africa, at concentrations of between 0.05 mg/L and 0.20 mg/L [23], and is typically about 7% [23]. The unbiodegradable soluble COD fraction is unaffected throughout the biological nutrient removal (BNR) process, thus it remains constant [13]. The readily biodegradable COD has been reported to consist of VFA and fermentable organic matter [24]. The settled sewage has been found to contain a high percentage of biodegradable particulate COD [13]. During the fermentation process these particulate biodegradable CODs have been reported to be hydrolysed into soluble compounds by bacteria while the soluble compounds are utilised by the bacteria under anaerobic conditions for the production of VFAs [25]. Thus the efficiency of the fermentation process will be reflected by an increase in the SCOD concentration in the process. 

**Figure 3:**
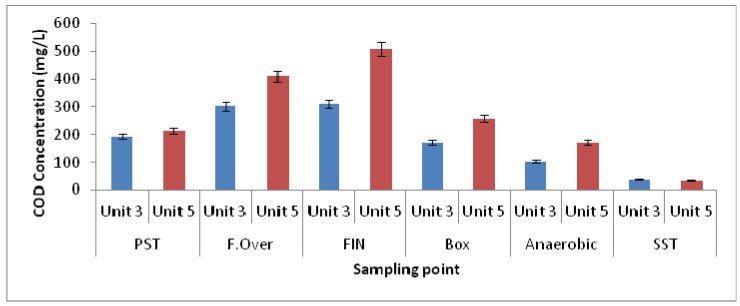
COD variations in wastewater samples collected from different zones of both Unit-3 and Unit-5 of the Northern Wastewater Treatment Works, Johannesburg, South Africa.

The SCOD concentrations from the settled sewage (overflow from PST) yielded averages of 192.8 mg COD/L for Unit-3 and 214.6 mg COD/L for Unit-5, and increased to 301.8 mg COD/L and to 411.6 mg COD/L respectively in the overflow from the elutriation tanks of the two units (Figure 3). The side-streams from the elutriation tanks are pumped into the balancing tank where mixing takes place with the settled sewage from the PSTs. This operation reduces the SCOD concentrations, as can be seen in the results of the analysis of the samples taken from the division box, namely 170.9 mg COD/L and 256.3 mg COD/L for Unit-3 and Unit 5, respectively (Figure 3). 

Thus, in the case of Unit-3, the SCOD concentration in the overflow from the division box, from the balancing tank, was similar to that of the settled sewage from the PSTs, which indicates that most of the generated SCOD was diluted in the balancing tank. Samples taken inside the elutriation tanks of the two units revealed that Unit-5 generated more SCOD, namely 508.2 mg COD/L, compared to that of Unit-3, which generated 312.0 mg COD/L. The results obtained for the elutriation tank in Unit-5 are comparable to those obtained by Bouzas *et al.* [26]. These authors performed their experiments in a pilot-scale side-stream fermenter at varying sludge ages, recirculation sludge flow rates and waste sludge flow rates. For sludge ages of between 4 days and 6 days the SCOD concentrations were reported to vary between 353 mg/L and 720 mg/L.

**Table 3:** Concentrations of volatile fatty acids in the samples collected from the elutriation tanks (Unit-3 and Unit-5), Northern Wastewater Treatment Works, Johannesburg, South Africa.

	Elutriation Tank Unit-5	Elutriation Tank Unit-3	Elutriation Tank Unit-5	Elutriation Tank Unit-3
	2012/04/12		2012/07/12	
**VFA**	**Conc. (mg/L)**	**Conc. (mg/L)**	**Conc. (mg/L)**	**Conc. (mg/L)**
Hexanoic acid, methyl ester	ND	ND	ND	ND
Heptanoic acid, methyl ester	ND	ND	ND	ND
Butanoic acid, 3-methyl-	ND	17.38 ± 0.87	67.41 ± 3.37	56.34±2.82
Pentanoic acid, 4-methyl-	ND	13.64 ± 0.68	ND	ND
Acetic acid	57.65 ± 2.88	26.32 ± 1.32	58.85 ± 2.94	54.98 ± 2.75
Propanoic acid	38.61 ± 1.93	17.19 ± 0.86	44.45 ± 2.22	43.56 ± 2.18
Isobutyric acid	57.27 ± 2.86	29.67 ± 1.48	56.39 ± 2.82	43.96 ± 2.20
Butanoic acid	73.13 ± 3.66	49.68 ± 2.48	66.08 ± 3.30	50.67 ± 2.53
Pentanoic acid	76.6 ± 3.83	87.6 ± 4.38	69.45 ± 3.47	61.58 ± 3.08
Heptanoic acid	88.52 ± 4.43	33.98 ± 1.70	89.82 ± 4.49	53.97 ± 2.70
	2012/05/14		2012/08/13	
Hexanoic acid, methyl ester	ND	ND	ND	ND
Heptanoic acid, methyl ester	21.63 ± 1.08	ND	ND	ND
Butanoic acid, 3-methyl-	67.41 ± 3.37	37.95 ± 1.90	67.41±3.37	53.69±2.68
Pentanoic acid, 4-methyl-	80.15 ± 4.01	38.23 ± 1.91	ND	ND
Acetic acid	58.85 ± 2.94	39.68 ± 1.98	54.05±2.70	41.29±2.06
Propanoic acid	43.71 ± 2.19	43.32 ± 2.17	49.63±2.48	34.67±1.73
Isobutyric acid	57.27 ± 2.89	23.14 ± 1.16	56.39±2.82	43.95±2.20
Butanoic acid	65.2 ± 3.26	16.52 ± 0.83	70.49±3.52	61.84±3.09
Pentanoic acid	69.45 ± 3.47	12.65 ± 0.63	77.62±3.88	81.39±4.07
Heptanoic acid	92.39 ± 4.62	39.37 ± 1.97	ND	3.96± 0.20
	2012/06/12			
Hexanoic acid, methyl ester	ND	ND		
Heptanoic acid, methyl ester	ND	ND		
Butanoic acid, 3-methyl-	67.41±3.37	43.39±2.17		
Pentanoic acid, 4-methyl-	ND	ND		
Acetic acid	57.65±2.88	45.25±2.26		
Propanoic acid	43.71±2.19	36.54±1.83		
Isobutyric acid	55.51±2.78	21.58±1.08		
Butanoic acid	65.2±3.16	23.95±1.20		
Pentanoic acid	69.45±3.26	46.39±2.32		
Heptanoic acid	91.13±4.56	85.34±4.27		

Note: ND: Not detectable; Conc.: Concentration.

Analysis of the VFA concentrations in samples collected from the elutriation tanks showed that the following short-chain volatile fatty acids (SCVFA) were present in the elutriation tank: Acetic acid, propionic acid and isobutyric acid at concentrations ranging between 38.61 mg/L and 58.85 mg/L and between 17.19 mg/L and 54.98 mg/L in Unit-5 and Unit-3, respectively (Table 3). In addition to the SCVFAs, the long-chain volatile fatty acids (LCVFA) such as heptanoic acid, 3-methylbutanoic acid, pentanoic acid, 4-methylpentanoic acid, and methylheptanoic acid were also present at high concentrations, ranging from 21.63 mg/L to 92.39 mg/L and from 3.96 mg/L to 85.34 mg/L for Unit-5 and Unit-3, respectively. These values were found to be higher than those reported in the literature [8]. By comparing the two units, it shows that the elutriation tank of Unit-5 contained more VFAs than the elutriation tank of Unit-3 and this could be due to the fact that the elutriation tank in Unit-5 is covered, whereas it is open in Unit-3. The sample collected in May (14 May 2012) from Unit-5 contained the highest concentration of acetic acid (58.85 mg/L), propanoic acid (43.71 mg/L) and isobutyric acid (57.27 mg/L); while the sample collected in July from Unit-3 contained the highest SCVFAs. The SCVFAs have been reported to enrich the EBPR system with polyphosphate-accumulating organisms, resulting in greater phosphate-removal efficiency [10]. Usually, the most dominant VFA is acetate, followed by butyrate and propionate [27]. However, during the sampling cycle of the present study for Unit-3 (the samples collected in April, May and June), propionate was found to be the second most dominant VFA. This disturbance could not be explained. Longer-chain VFAs, such as butyric, isobutyric, valeric and isovaleric acids, have received little attention, primarily because they are either completely non-detectable or they are present in low concentrations in a properly functioning digester [28,29]. However, in the present study, LCVFAs were found in higher concentrations than the SCVFAs. 

During the course of the study, phosphate release did occur in the anaerobic zones of both units as the phosphate concentration increased from 4.28 mg P/L (settled sewage from PSTs) to 9.62 mg P/L in the anaerobic zone of Unit-3 (Bioreactor 1) and similarly from 3.85 mg P/L to 9.69 mg P/L in Unit-5. Thus, good phosphate release was observed in both units, although the SCOD concentration in Unit-5 was slightly higher than that of Unit-3 in Bioreactor 1. 

**Figure 4:**
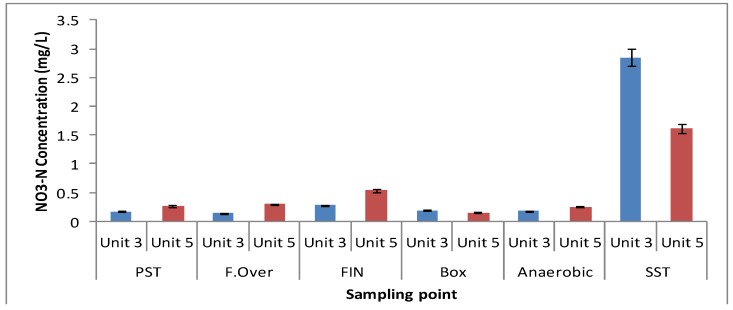
Nitrate concentration in wastewater samples collected from different zones of both Unit-3 and Unit-5 of the Northern Wastewater Treatment Works.

**Figure 5:**
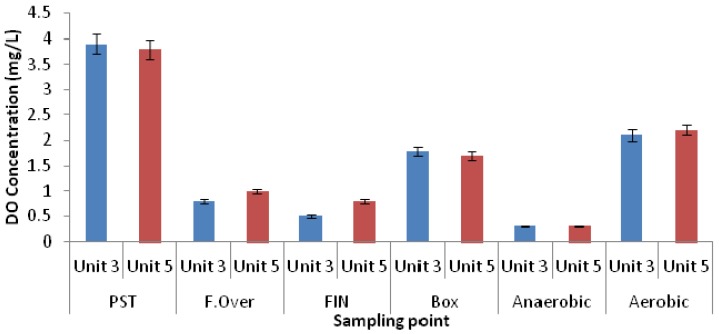
DO concentration in wastewater samples collected from different zones of both Unit-3 and Unit-5 of the Northern Wastewater Treatment Works.

The nitrate concentrations throughout the different zones in both units were found to be low, as seen in Figure 4. On average, a DO concentration of 0.3 mg/L was recorded in the anaerobic zones of both units (Figure 5). As the phosphate release corresponded to the required limits in both units, the readily biodegradable COD in both units was sufficient to sustain the PAO organisms in order to release phosphate. 

Thus, taking into account the finding that the performance of Unit-5 was slightly better than that of Unit-3, it would suggest that the phosphate uptake in Unit-5 was slightly higher than that of Unit-3. One important factor which had an influence on the phosphate uptake in the aerobic zone was the amount of oxygen available. The average DO concentrations in the aerobic zones of Unit-3 (Bioreactor 1) and Unit-5 were 2.1 mg/L and 2.2 mg/L, respectively, which indicated that the DO concentration in both units fell within the same range (Figure 5). It is known that oxygen is required to remove COD for nitrification and to achieve enhanced phosphate uptake [23,30]. The oxygen demand to satisfy the requirement for COD removal has been reported to be related to the mass of sludge in the reactor, as well the sludge age [23].

It was observed that the MLSS concentration in Unit-5 was slightly lower, namely 2,058 mg/L, compared to the MLSS concentration of 2,485 mg/L in Unit-3; thus the sludge mass in Unit-5 was slightly lower than the sludge mass in Unit-3. Further, the amount of sludge wasted from both units (Unit-5 sludge wasting rate: 2,074 m^3^/d, Unit-3 sludge wasting rate: 3,888 m^3^/d) was based on the MLSS concentration and ammonia concentrations in the effluent. Due to insufficient data on TKN concentrations, the oxygen requirements for nitrification, which can be calculated from the nitrification capacity (which is the difference between influent and effluent TKN concentrations and the amount of nitrogen used to form new biomass), could not be estimated. The bacterial oxidation of ammonia to nitrate also requires oxygen. A comparison of the ammonia concentrations in the effluents from Unit-3 and Unit-5 indicated that the nitrification in Unit-3 was slightly better than that in Unit-5 (0.72 mg∙N/L and 1.28 mg∙N/L, respectively). Thus, as the volumes of both units were estimated to be the same and the concentrations in the underflow were 2 × MLSS in the reactors, the sludge ages of the two units were estimated to be 10 days for Unit-5 and 5 days for Unit-3. However, it should be mentioned that the sludge age of 5 days for Unit-3 was not found to be realistic due to uncertainty regarding the data. Over-aeration can also have a negative impact on phosphate uptake.

**Figure 6:**
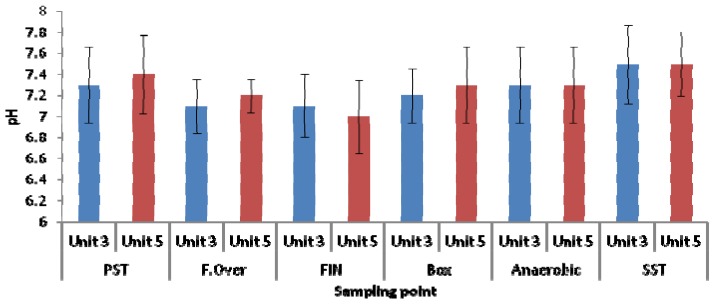
pH variation in wastewater samples collected from different zones of both Unit-3 and Unit-5 of the Northern Wastewater Treatment Works.

As depicted in Figure 6, in general, the pH values in all the zones ranged from pH 7 to pH 7.5 for both units. It has been reported that most of the bacteria grow at pH ranges of between 6 and 8. A study conducted by Tracy and Flammino [31] reported that EBPR mechanisms do not function at a pH value of less than 5.4. However, at pH values ranging from 8.5 to 9, the EBPR mechanisms can still operate, but in concomitance with high chemical precipitation of phosphate.

When studying the relationship between the amounts of orthophosphate released in EBPR and the acetate taken up under anaerobic conditions on the one hand and the pH of the system on the other hand, Smolders *et al.* [32] reported that the transport of acetate through the cell membrane is energy-dependent and the amount of energy needed is pH-dependent. In addition, Liu *et al.* [33] also reported that at a pH value of below 5, no acetate uptake was observed, while in the range of pH 5 to pH 6.5 acetate uptake increased significantly (0 to approximately 50 mg∙C/g∙VSS∙h). These authors also observed that within the pH range of pH 6.5 and pH 8, the acetate uptake is not pH-dependent, but outside this range, at above pH 8, the uptake starts to decrease significantly. Schuler and Jenkins [34] also reported that the glycogen/acetate uptake in the EBPR decreases with an increase in pH to above pH 7. The said author furthermore pointed out that at a pH of above 7 the polyphosphate-accumulating organisms have a competitive advantage over the glycogen-accumulating organisms, in terms of their respective metabolic processes. In contrast, US-EPA [35] reported that nitrification and phosphorus removal rates decrease when pH levels drop to below 6.9 and glycogen-accumulating organisms may also compete with the polyphosphate-accumulating organisms at pH levels of less than pH 7. When investigating the effect of pH on nitrification and denitrification, Glass *et al.* [36] reported that at pH values of 6 to 7, and nitrite concentrations of 30 mg/L NO_2_-N and 250 mg/L NO_2_-N, respectively, could inhibit denitrification. The pH is therefore an important factor determining the performance of an enhanced biological phosphorus removal process in wastewater treatment systems.

### 3.2. Microbial Ecology

**Figure 7:**
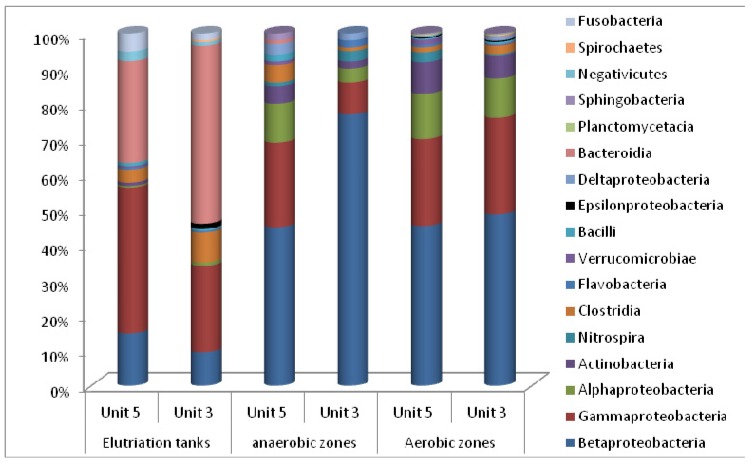
Microbial diversity (by phylum) of the Northern Wastewater Treatment Works (Unit-3 and Unit-5), Johannesburg, South Africa.

At present, there is a particular interest in the relationship between biodiversity, simply defined as the number of species present in the system, and their function in the wastewater treatment works. In this study, the biodiversity of the Johannesburg Northern Wastewater Treatment Works was assessed. The microbial community structure of the wastewater samples was determined using the 16S rRNA gene amplicon pyrosequencing method which targeted one DNA region (V1-3); respective sequences are summarised in Table 1. A total of approximately 2,000 sequences (Table 3) were identified in the wastewater and only sequences with a similarity of 95% to 100% were used. To determine the abundance of each taxon, a plot was generated of the number of sequences of a particular taxon against the total number of sequences used. The bacterial phyla that were identified in the activated sludge collected from the Northern Wastewater Treatment Works in both Unit-5 and Unit-3 are illustrated in Figure 7. The microbial populations of Unit-5 (13 classes from the aerobic zone, 11 classes from the anaerobic zone and 11 from the elutriation tanks) appeared to be slightly more diverse compared to those of Unit-3 (13 classes from aerobic zone, 8 classes from anaerobic zone and 11 from the elutriation tanks) in terms of bacterial classes. The sequencing results indicated that in Unit-5 over 75% of the total microbial population was occupied by Gammaproteobacteria (41.4%), Bacteroidia (28.84%), and Betaproteobacteria (14.86%) in the elutriation tanks; by Betaproteobacteria (45%), Gammaproteobacteria (24%) and Alphaproteobacteria (11%) in the anaerobic zones; and by Betaproteobacteria (45.36%), Gammaproteobacteria (24.64%) and Alphaproteobacteria (12.86%) in the aerobic zones. A similar observation was noted in the bioreactor of Unit-3 (the anaerobic and aerobic zones) where Betaproteobacteria (77%), Gammaproteobacteria (9%) and Alphaproteobacteria (4%) were found in the anaerobic zones, and Betaproteobacteria (48.61%), Gammaproteobacteria (27.43%) and Alphaproteobacteria (11.11%) in the aerobic zone. 

It has frequently been reported that the GAOs are members of the Alpha- and Gammaproteobacteria and Actinobacteria [14]. In this study, Betaproteobacteria were dominant in anaerobic and aerobic zones in both the units, whereas Gammaproteobacteria were observed to be the dominant class in the elutriation tanks of Unit-5 followed by Bacteroidia*,* and in Unit-3, Bacteroidia was more dominant than Gammaproteobacteria. The difference in the relative abundance between the microbial population in the elutriation tanks of Unit-5 and Unit-3 could be due to the structure of the tanks because covering Unit-5 allowed the predominance of Gammaproteobacteria instead of Bacteroidia as in Unit-3. In addition, Alphaproteobacteria were less abundant in both Unit-5 and Unit-3 with a high abundance in the aerobic zones when compared to the anaerobic zones and elutriation tanks.

**Figure 8:**
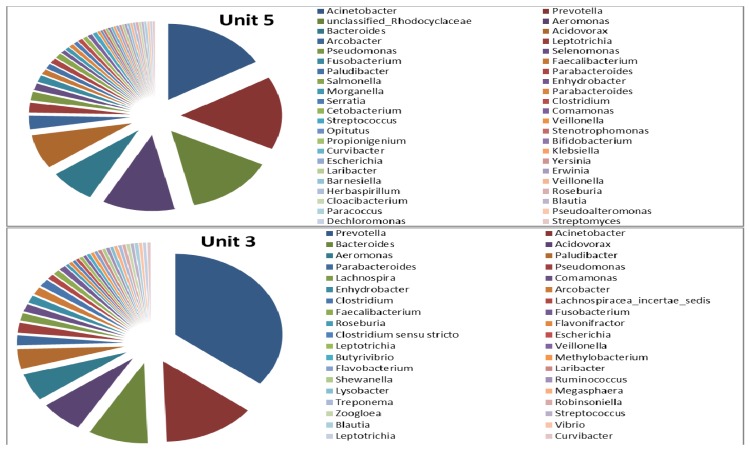
Microbial diversity (by genus) of elutriation tanks in Unit-5 and Unit-3 of the Northern Wastewater Treatment Works, Johannesburg, South Africa.

**Figure 9:**
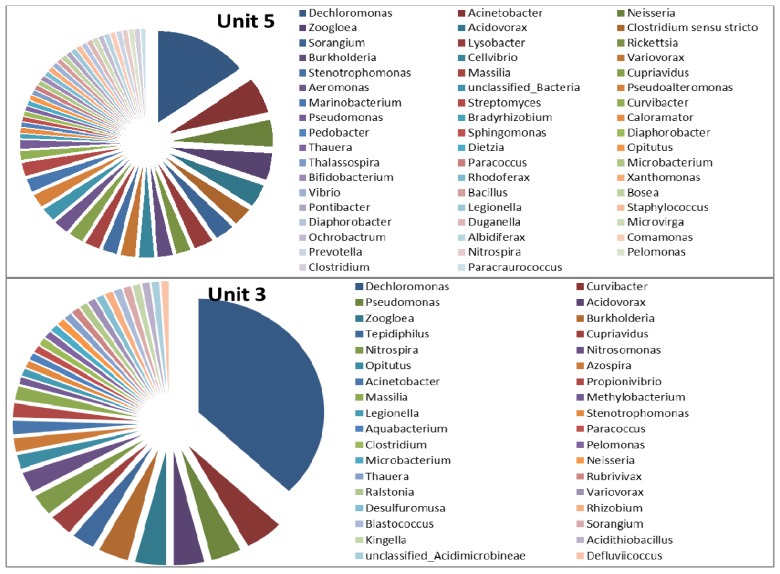
Microbial diversity (by genus) of anaerobic zones in Unit-5 and Unit-3 of the Northern Wastewater Treatment Works, Johannesburg, South Africa.

**Figure 10:**
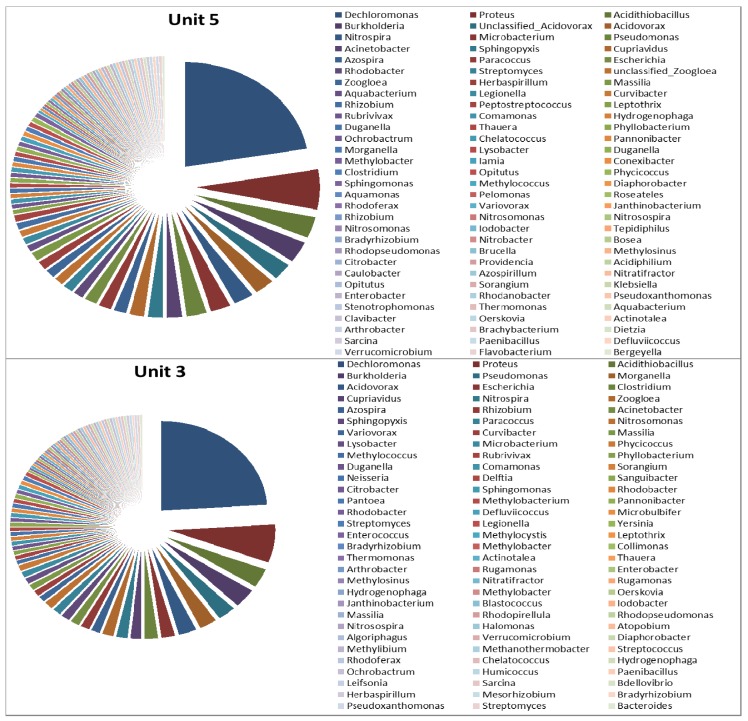
Microbial diversity (by genus) of aerobic zones in Unit-5 and Unit-3 of the Northern Wastewater Treatment Works, Johannesburg, South Africa.

Genus-level identification of the bacterial populations revealed a much more diverse microbial population in Unit-5 than in Unit-3 (Figure 8, Figure 9 and Figure 10). In the elutriation tanks, a large abundance of uncultured microorganisms was observed in Unit-5, comprising approximately 44 genera with *Acinetobacter* (16.67%), *Prevotella* (15.58%), unclassified-*Dachloromonas* (14.13%), *Aeromonas* (11.59%), *Bacteroides* (7.25%), *Acidovorax* (7.25%), *Arcobacter* (2.9%) and *Leptotrichia* (2.17%) as the most predominant genera, representing approximately 78% of the total microbial population. The rest of the microbial population detected in the elutriation tanks of Unit-5 represented 22% of the total microbial population with a percent abundance of less than 2% each. In the elutriation tank of Unit-3, the microbial diversity was comprised of approximately 38 genera with *Prevotella* (35.5%), *Acinetobacter* (14%), *Bacteroides* (9%), *Acidovorax* (6.5%), *Aeromonas* (5.5%), *Paludibacter* (4%), *Parabacteroides* (2%) and *Pseudomonas* (2%) representing approximately 78.5% of the total microbial population. The remaining 21.5% of the microbial population comprised more than 30 genera with an abundance of less then 2% each. 

As is evident in Figure 9, in the anaerobic zones (of the bioreactors), the microbial population of Unit-5 appeared to be more diverse than that of Unit-3 with approximately 53 and 36 genera, respectively. In Unit-5, 20 microbial genera represented approximately two thirds of the microbial population with the percent abundance of *Dechloromonas*, *Acinetobacter*, *Neisseria*, *Zoogloea*, *Acidovorax*, *Clostridium*, *Sorangium* and *Lysobacter* being 14.96%, 6.3%, 4.72%, 4.72%, 3.94%, 3.15%, 3.15%, and 3.15%, respectively. In the anaerobic zone of Unit-3, nine microbial genera represented approximately two thirds of the total microbial population with the percent abundance of *Dechloromonas*, *Curvibacter*, *Pseudomonas*, *Acidovorax*, *Zoogloea*, *Burkholderia*, *Tepidiphilus*, *Cupriavidus*, *Nitrospira* and *Nitrosomonas* being approximately 37.25%, 4.9%, 3.92%, 3.92%, 3.92%, 3.92%, 2.94%, 2.94%, 2.94% and 2.92 %, respectively. The remaining 30% of the microbial population comprised more than 27 microbial genera, each with an abundance of less than 2%. 

In the aerobic zones of the bioreactors, the results revealed no significant difference in terms of microbial diversity between Unit-5 and Unit-3 with a large number of microbial genera, that is 92 and 93 genera, respectively (Figure 10). In the aerobic zone of Unit-5, 31 of the 92 genera and nine of the 92 genera represented approximately 75% and 50% of the total microbial population, respectively. Of those microbial genera, *Dechloromonas* (22.37%) was reported to be the most predominant, followed by *Proteus* (6.25%), *Acidithiobacillus* (3.29%), *Burkholderia* (3.29%), unclassified *Acidovorax* (2.63%), *Acidovorax* (2.63%), *Nitrospira* (2.63%), *Microbacterium* (2.63%), and *Pseudomonas* (2.63%), with the remaining genera forming less than 2% of the total bacterial population. A similar observation was noted in the aerobic zone of Unit-3, where nine out of 93 genera and 33 out of 93 genera represented approximately 50% and 75% of the total microbial population. Among these, *Dechloromonas* (23.97%) was reported to be the most predominant, followed by *Proteus* (6.85%), *Acidithiobacillus* (3.42%), *Burkholderia* (3.42%), *Pseudomonas* (2.74%), *Morganella* (2.74%), *Acidovorax* (2.74%), *Eschirichia* (2.05), and *Clostridium* (2.05%), with the remaining genera forming less than 2% of the total bacterial population. 

As determined by culture-dependent methods, bacteria belonging to the genus *Acinetobacter* were long believed to be potential PAOs and to play a primary role in phosphate removal in a full-scale EBPR [37]. However, recent studies in which culture-independent 16S rRNA-based molecular techniques were used, including fluorescence *in situ* hybridisation (FISH), revealed that this is not the case [6,10]. As a substitute, several authors have proposed that *Rhodocyclus*-related bacteria are important PAOs [38,39]. Furthermore, Wagner *et al.* [40] pointed out that it was with the use of specific FISH probes that *Acinetobacter* was shown to have little significance in full-scale plants when compared to members of other phylogenetic groups such as the Betaproteobacteria and Actinobacteria [40]. However, *Acinetobacter* was found in abundance in the elutriation tanks of Unit-5, while in Unit-3 it was the second most abundant genus and could be responsible for the production of VFAs in the elutriation tanks. Furthermore, when characterising the EBPR using DGGE, Ren *et al.* [41] also reported *Acidovorax* spp. as one of the most dominant species and the isolates responsible for phosphate removal. However, the presence of *Rhodocyclus*-related bacteria belonging to the family Rhodocyclaceae, such as *Dechloromonas* spp., *Zoogloea* spp. and *Thauera* spp., was observed in relative abundance in the anaerobic and aerobic zones of both Unit-5 and Unit-3, compared to the other microbial groups. In addition, bacteria belonging to *Acidovorax* spp. were also found in the aerobic and anaerobic zones in both units. As reported by Ren and co-authors [41], the PAOs in the activated sludge are not a sole species but are considered to be a group of microorganisms able to accumulate phosphate in high concentrations. Therefore, *Proteus*, *Acidithiobacillus*, *Burkholderia*, and other genera found to be highly abundant could be involved in the removal of phosphate in the EBPR. Despite the abundance of *Rhodocyclus*-related organisms in the EBPR, and their association as primary PAOs, possibly new species belonging to the genera of *Azospira* and *Azovibrio* and *Thauera* and *Zoogloea* were recently identified and are also considered to be potential PAOs [42]. In addition, the function of genus *Arcobacter* previously found in significant numbers in activated sludge has been undetermined [43]. In a study conducted by Mullan *et al.* [44] it was pointed out that bacteria belonging to the family Burkholderiaceae such as *Burkholderia cepacia* were revealed to possess maximum phosphate-removal ability while also being able to accumulate polyphosphate at pH 5.5. Although the microbial diversity of the two units was slightly different with the most diverse microbial population being found in Unit-5, this difference was also revealed in the nutrient-removal efficiency during the process (Figure 2 and Figure 3).

Glycogen-accumulating organisms (GAOs) are often associated with a sudden breakdown of the EBPR systems by outcompeting PAOs for volatile fatty acids [45]. 

Regardless of the *candidatus competibacter phosphatis*, it has been reported that the *Defluviicoccus* spp. is also one of the most important GAOs in wastewater treatment systems [46]. Beer *et al.* [47] have also reported that the precise identity of these GAOs is still largely unknown. In the present study, GAOs were found in the aerobic zones of both units at a percentage of 0.68% (*Defluviicoccusngomonas*) for Unit-3, and at 0.33% (*Defluviicoccus*) for Unit-5. Another microbial genus, namely *Sphingopyxis* belonging to the family of Sphingomonadaceae, was also found at a percentage of 1.32% in Unit-5 and 1.37% in Unit-3. In addition, the GAO populations were also found in anaerobic zones of Unit-3 at a rate of 9.8% (*Defluviicoccus*).

In the present study, denitrifying representatives of Alpha-, Beta-, Gamma-, and Epsilonproteobacteria, Firmicutes, and Bacteroidetes were found, as also reported by Heylen *et al.* (2006) [42]. The genera classically known as denitrifiers such as *Pseudomonas*, *Ochrobactrum* and *Acidovorax* were also encountered in both Unit-5 and Unit-3. This is in agreement with the findings of Schramm *et al.* [48] who reported that at least 16 species from two genera belonging to the *Betaproteobacteria*, namely *Nitrosomonas* (formerly *Nitrosococcus* *mobilis* and *Nitrosomonas*) and *Nitrosospira* (formerly *Nitrosospira*, *Nitrosovibrio* and *Nitrosolobus*) have been shown to be able to perform ammonia oxidation, while another four genera, namely *Nitrobacter*, *Nitrospina*, *Nitrococcus* and *Nitrospira*, have been shown to be able to perform nitrite oxidation. Despite the diversity of ordinary heterotrophic organisms (OHO) present in both units, this did not affect the performance of the plants because of their relatively low numbers. 

## 4. Conclusions

The EBPR process of Northern Wastewater Treatment Works in its Unit-5 and Unit-3 was investigated in terms of their physicochemical and microbiological profile. High-quality effluents were produced in both units in terms of phosphate-removal efficiency (of 0.08 mg P/L for Unit-5 and 0.11 mg P/L for Unit-3) throughout the study period. The VFAs, COD concentrations and the pH values observed in the elutriation tanks as well as in the bioreactors (aerobic and anaerobic zones) of both units were found to be suitable for an excellent PAO growth performance, although Unit-5 appeared to function much better than Unit-3 did. As indicated by the pyrosequencing analysis, a significant microbial community was observed in Unit-5 compared to Unit-3. The results indicated that the most dominant sequences from both units belonged to the Gammaproteobacteria, Bacteroidia, Betaproteobacteria and Alphaproteobacteria. The good EBPR performance was positively associated with the high presence of PAOs in both units. Glycogen-accumulating organisms (GAOs) were detected only in the aerobic zones (bioreactors) at relatively low rates in both Unit-5 and Unit-3. 

During the course of this study, it became evident that several areas in this field require further research. The following information is required not only for a better understanding of the microbial ecology of the EBPR process, but also for effective phosphate removal in full-scale EBPR plants. As the PAO and GAO populations in EBPR systems now appear to be phylogenetically diverse, more studies of these physiological groups are needed to clarify their diversity and to further elucidate their ecophysiology. As a result, novel molecular techniques such as metagenomics and biochemical characterisation should be continuously developed and employed in order to directly link the substrate uptake and carbon polymer storage (PHA or others) with glycogen consumption under anaerobic conditions. Full-scale activated sludge systems often deal with a wide range of organic matter including carboxylic acids, sugars, and amino acids. The importance of carbon sources other than VFAs for the proliferation of PAOs or GAOs is not clear at present. Investigation of the metabolism of organic substrates should thus not be limited to short-chain VFAs only, but should be expanded to include long-chain VFAs as well as other carbon sources. 
